# A cross-tissue transcriptome-wide association study identifies new susceptibility genes for frailty

**DOI:** 10.3389/fgene.2024.1404456

**Published:** 2024-07-12

**Authors:** Daoyi Lin, Shuyan Wu, Wangyu Li, Peng Ye, Xuan Pan, Ting Zheng, Fei Gao

**Affiliations:** ^1^ Department of Anesthesiology, Fujian Provincial Hospital, Shengli Clinical Medical College of Fujian Medical University, Fuzhou University Affiliated Provincial Hospital, Fuzhou, China; ^2^ Department of Anesthesia, China-Japan Friendship Hospital, Chinese Academy of Medical Sciences and Peking Union Medical College, Beijing, China; ^3^ Department of Pain Management, Shengli Clinical Medical College of Fujian Medical University, Fujian Provincial Hospital, Fuzhou University Affiliated Provincial Hospital, Fuzhou, China; ^4^ Fujian Provincial Key Laboratory of Emergency Medicine, Fujian Emergency Medical Center, Fuzhou, China; ^5^ Fujian Provincial Co-Constructed Laboratory of “Belt and Road”, Fujian Emergency Medical Center, Fuzhou, China

**Keywords:** frailty, TWAS, UTMOST, mendelian randomization, causal relationship

## Abstract

**Background:** Although genome-wide association studies (GWAS) have identified 14 loci associated with frailty index (FI) susceptibility, the underlying causative genes and biological mechanisms remain elusive.

**Methods:** A cross-tissue transcriptome-wide association study (TWAS) was conducted utilizing the Unified Test for Molecular Markers (UTMOST), which integrates GWAS summary statistics from 164,610 individuals of European ancestry and 10,616 Swedish participants, alongside gene expression matrices from the Genotype-Tissue Expression (GTEx) Project. Validation of the significant genes was performed through three distinct methods: FUSION, FOCUS, and Multiple Marker Analysis of Genome-wide Annotation (MAGMA). Exploration of tissue and functional enrichment for FI-associated SNPs was conducted using MAGMA. Conditional and joint analyses, along with fine mapping, were employed to enhance our understanding of FI’s genetic architecture. Mendelian randomization was employed to ascertain causal relationships between significant genes and FI, and co-localization analysis was utilized to investigate shared SNPs between significant genes and FI.

**Results:** In this study, two novel susceptibility genes associated with the risk of FI were identified through the application of four TWAS methods. Mendelian randomization demonstrated that HTT may elevate the risk of developing frailty, whereas LRPPRC could offer protection against the onset of frailty. Additionally, co-localization analysis identified a shared SNP between LRPPRC and FI. Tissue enrichment analyses revealed that genomic regions linked to SNPs associated with frailty were predominantly enriched in various brain regions, including the frontal cortex, cerebral cortex, and cerebellar hemispheres. Conditional, combined analyses, and fine mapping collectively identified two genetic regions associated with frailty: 2p21 and 4q16.3. Functional enrichment analyses revealed that the pathways associated with frailty were primarily related to the MHC complex, PD-1 signaling, cognition, inflammatory response to antigenic stimuli, and the production of second messenger molecules.

**Conclusion:** This investigation uncovers two newly identified genes with forecasted expression levels associated with the risk of FI, offering new perspectives on the genetic architecture underlying FI.

## 1 Background

Frailty (Dent et al., 2019; Hoogendijk et al., 2019) is recognized as a prevalent geriatric syndrome characterized by the increased vulnerability of older adults to stress due to declining physical function and reduced physiological reserve capacity. Contrary to aging, defined as a natural and irreversible process characterized by changes at physiological, molecular, and cellular levels, frailty represents a preventable and treatable condition, a phenotype of aging that, with prolonged exposure, escalates the risk of adverse outcomes including falls, disability, long-term care, and death (McIsaac et al., 2020; Shi et al., 2021), highlighting the critical significance and intrinsic complexity of this trait. The global prevalence of frailty among individuals aged ≥50 years is reported to be 24% (Nguyen et al., 2015; O'Caoimh et al., 2021), with its prevalence increasing with age, and women exhibiting a higher prevalence than men. Frailty is commonly quantified using the (FI) (Cesari et al., 2014; Kojima et al., 2018), which assesses an individual’s level of frailty by evaluating the number of deficits across a range of physiological parameters, diseases, disabilities, and health indicators. Although GWAS has pinpointed 14 genetic loci associated with FI, these variants account for only 11% of the FI’s heritability (Atkins et al., 2021). Currently, there are few studies on frailty from the perspective of genetics. A more comprehensive understanding of the genetic basis of frailty and the exploration of genes associated with frailty indices and their functions hold significant potential for promoting healthy aging by revealing the molecular mechanisms affecting the development of frailty, such as muscle degradation, reduced immune function, and increased inflammatory response (Ferrucci and Fabbri, 2018). This understanding can help to identify new therapeutic targets. Meanwhile, the current diagnosis of frailty mainly relies on relevant scales. However, scale assessment is somewhat subjective, and patients are often already in the clinical stage by the time of detection. Investigating the genetic basis of frailty can help identify new biomarkers to early and objectively recognise frailty, thus reducing the incidence of adverse outcomes.

Transcriptome-wide association studies (TWAS) (Wainberg et al., 2019) represent an approach that combines gene expression data with genomic association studies (GWAS) to identify statistical associations between specific phenotypes or disease states and gene expression levels. Through this approach, researchers are able to identify genes that may influence disease risk through altered expression, offering insights for subsequent functional validation and studies on biological mechanisms, even if the gene variants themselves are not directly associated with disease in conventional GWAS. The Unified Test for Molecular Signatures (UTMOST) (Hu et al., 2019), a tool for TWAS, analyzes and integrates multiple datasets across organizations, enhancing statistical power and is more adept at uncovering significant genetic associations that might have been missed in traditional single-organization or single-study research. FUSION (Mai et al., 2023) identifies genes potentially influencing the phenotype via their expression levels by utilizing publicly available gene expression data and GWAS summary statistics, which aids in unraveling the underlying molecular mechanisms and biological pathways of diseases, crucial for comprehending the disease’s biology and developing novel therapeutic strategies. FOCUS (Lu et al., 2022) constitutes an advanced TWAS method specifically designed to accurately delineate gene expression patterns associated with phenotypes, utilizing fine-mapping techniques, FOCUS aims to more precisely identify specific genes contributing to variations in disease risk, minimize false-positive findings, and enhance the interpretability of genetic signals compared to conventional TWAS methods. The Multi-marker Analysis of GenoMic Annotation (MAGMA) (de Leeuw et al., 2015) serves as a tool for genomic data analysis, conducting gene association analysis, gene set enrichment analysis, and tissue-specific analysis. By analyzing genetic data at the gene and gene set levels, MAGMA can assist in identifying genes and pathways as potential biomarkers or therapeutic targets. Recently, cross-tissue association analysis has become widely utilized to screen for candidate susceptibility genes for complex multi-system diseases, including cardiovascular diseases, autoimmune diseases, and a variety of cancers.

**FIGURE 1:**
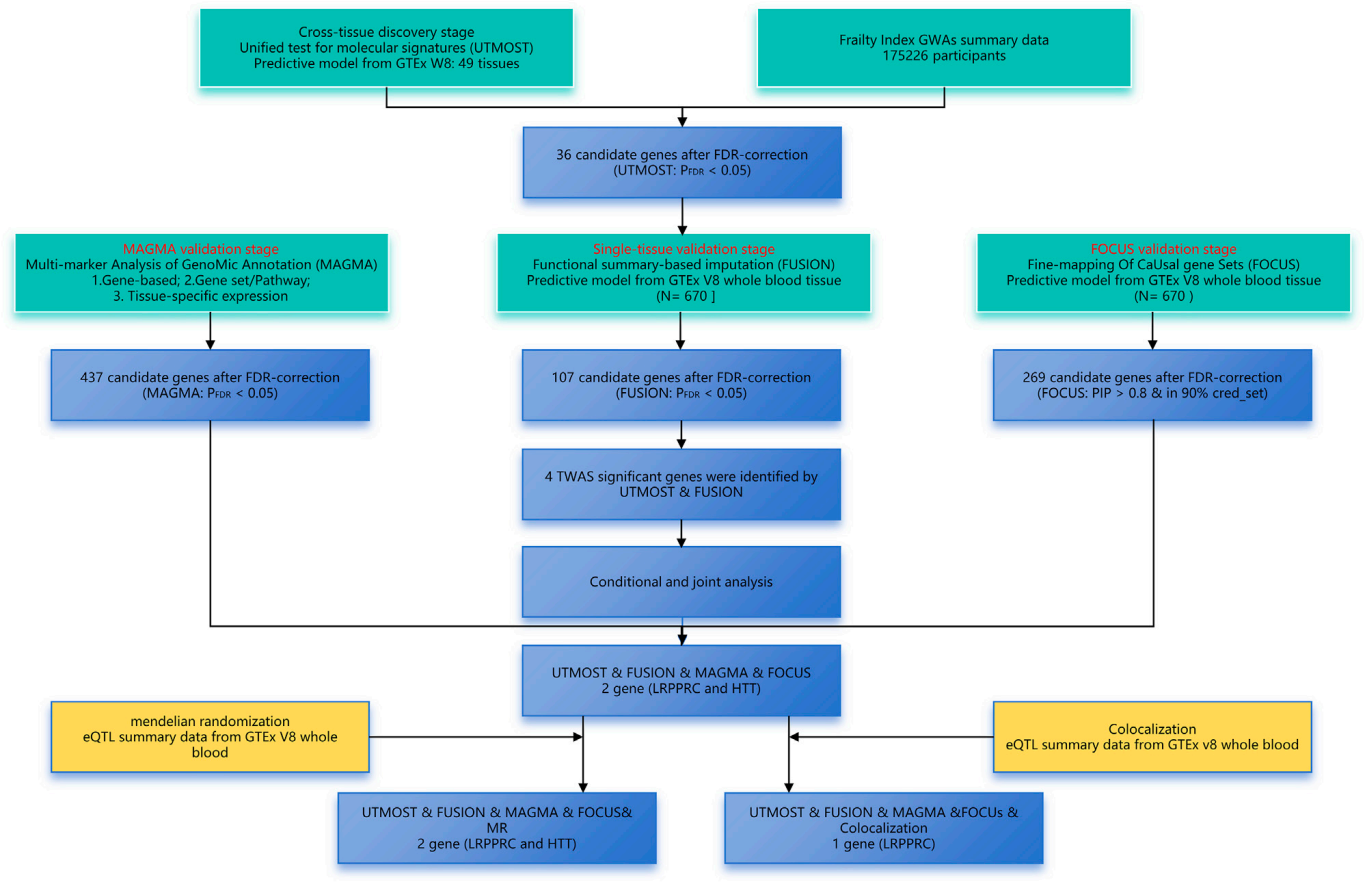
Overview of the transcriptome-wide association study design of FI. FDR, false discovery rate; GWAS, genome-wide association study; TWAS, transcriptome-wide association study.

In this work, we conducted a cross-tissue TWAS by integrating eQTL data from the Genotyped Tissue Expression (GTEx) project with the largest FI GWAS in Europe using the UTMOST method. We utilized three methods—FUSION, FOCUS, and MAGMA—to validate candidate susceptibility genes in parallel, employing conditional and joint analyses for genes independently associated with FI. Subsequent Mendelian randomisation and co-localisation analyses were conducted to further elucidate the relationship between genes and phenotypes. The detailed flowchart of these analyses is presented in Figure 1.

## 2 Materials and methods

### 2.1 FI GWAS data source

The GWAS data on frailty originated from the study conducted by Janice L. Atkins et al. (2021), representing the most comprehensive study to date on FI, including 164,610 individuals of European origin and 10,616 Swedish participants, all aged between 60 and 70 years. The FI was calculated using a cumulative assessment of deficits, referring to the proportion of an individual’s potentially unhealthy measures to all measures at a given point in time. It includes multidimensional health variables such as somatic, functional, psychological, and social variables, based on 49 self-reported items on symptoms, disabilities, and diagnosed illnesses. The FI ranges from 0 to 1, with higher values indicating higher levels of individual frailty. GWAS summary statistics for FI are available for download from the GWAS Catalog (study accession code GCST90020053).

### 2.2 TWAS analyses in cross-tissue and single tissue

First, inter-tissue association testing was explored using the UTMOST method. UTMOST (Hu et al., 2019; Rodriguez-Fontenla and Carracedo, 2021) can define gene-trait associations by considering SNP joint effects across linkage disequilibrium (LD) regions and integrating organismal GTEx data to create individual and cross-organisational covariance matrices. Next, we validated the results of UTMOST using three methods: FUSION, FOCUS, and MAGMA. FUSION (Gusev et al., 2016; Rodriguez-Fontenla and Carracedo, 2021) is a suite of tools for performing transcriptome-wide and regulator-wide association studies (TWAS and RWAS). It enables the creation of predictive models of the genetic component of a functional/molecular phenotype and the use of GWAS to summarise statistical predictions and test the association of that component with disease. FOCUS (Mancuso et al., 2019) assigns probabilities for interpreting observed association signals to each gene within a risk region by modelling correlations between TWAS signals. It is a probabilistic fine mapping method that prioritises genes with strong evidence of causality. MAGMA (Sey et al., 2023) first projects a gene’s SNP matrix onto its principal components, removes principal components with very small eigenvalues, and then uses these principal components as predictor variables for phenotype in linear regression models. This method is advantageous in the study of polygenic traits and in the exploration of the functional and biological mechanisms behind the genetic components of traits. Combining these complementary methods enhances the reliability of the results. Differences were deemed significant upon employing the Benjamini-Hochberg (B-H) correction method and establishing an FDR threshold below 0.05.

### 2.3 Conditional and joint analyses

In this study, conditional and association analyses were performed using FUSION software. Initially, the linkage disequilibrium (LD) matrices between SNPs were calculated for the genotype data and automatically generated by FUSION software based on the data from the 1,000 Genomes Project. Conditional analyses were performed through the assoc_test function of the FUSION software. Conditional analyses (Byrne et al., 2021) are mainly used in GWAS to identify multiple genetic variants with independent effects, which can help to distinguish whether the effect is the result of a single SNP or multiple SNPs working together, and determine the presence of other variants independently affecting the risk of disease. Joint analyses are performed through the joint_test function of the FUSION software. Joint analyses (Deng and Pan, 2018) are particularly suitable for the detection of small effect variants and improve the identification of rare variant associations.

### 2.4 Tissue-specific and pathway enrichment analysis

Using MAGMA, analyses for tissue-specific enrichment and gene set enrichment were conducted (de Leeuw et al., 2015). By integrating gene expression data (e.g., from the GTEx project), MAGMA can assess the expression patterns of genes across various tissues and determine their correlation with specific phenotypes. MAGMA can identify pathways or functional classes that are strongly associated with a disease or phenotype, by evaluating the collective performance of a specific gene set (e.g., genes within a certain pathway) in an association analysis.

### 2.5 Colocation analysis

Colocation analysis is a statistical method utilized to ascertain whether signals shared across two or more genetic study result sets (e.g., GWAS summary statistics from different diseases or phenotypes) originate from the same genetic variant. Colocation analyses were conducted using coloc. Coloc uses a Bayesian algorithm to generate posterior probabilities for five mutually exclusive hypotheses regarding shared causal variants in genomic regions, namely H0 (no association), H1 or H2 (associated with only one trait), H3 (associated with two traits, two different SNPs), and H4 (associated with two traits, one shared SNP). If the calculated posterior probabilities of H4 and H3 (PPH4 and PPH3) are greater than 0.5, the locus is considered co-localised.

### 2.6 Mendelian randomization

To explore the potential causal link between significant genes and frailty, Mendelian randomization analyses were carried out on the eQTL data for genes and the GWAS data for frailty. These analyses used inverse variance weighting (IVW) (Burgess et al., 2017) and employed the “TwoSampleMR” R packages, focusing on associations indicative of a causal relationship (*p* < 0.05).

## 3 Results

### 3.1 Transcriptome-wide association study results of FI

**FIGURE 2:**
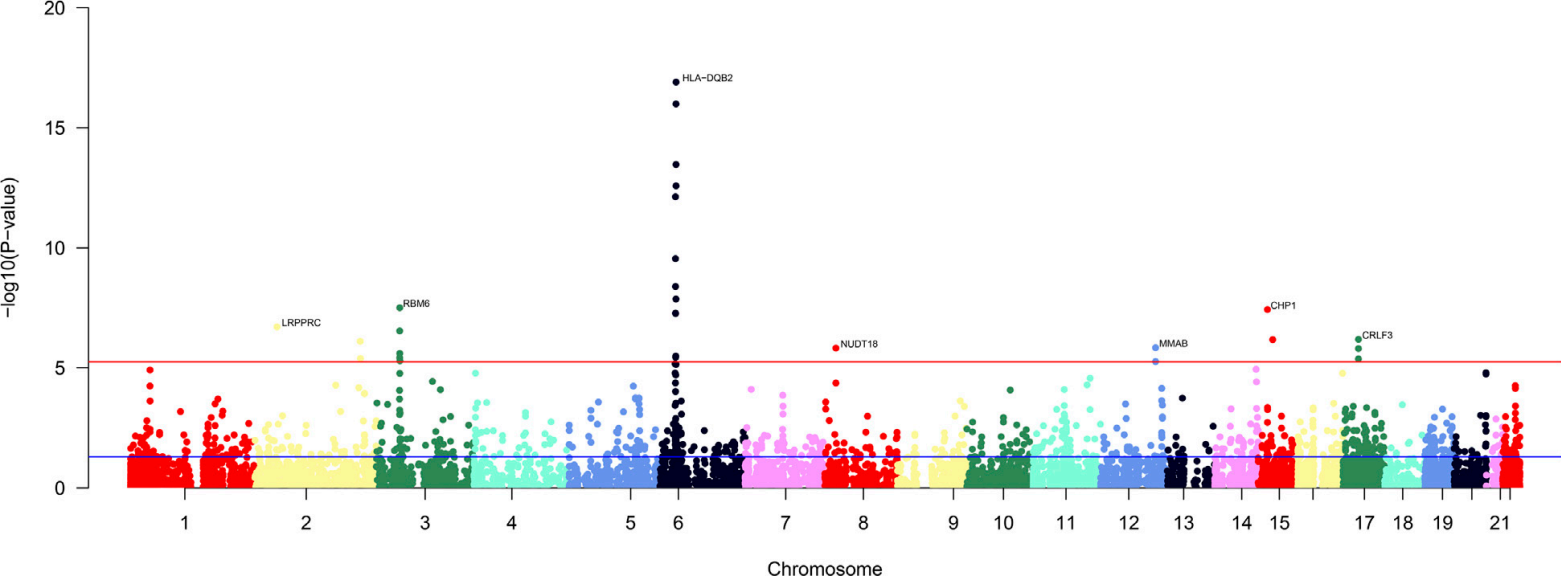
Manhattan plot of the cross-tissue transcriptome-wide association results for FI. 107 genes was specifically associated with the risk of FI. The *y*-axis represents *p*-value in –log(10) scale. A significance threshold after FDR-correction was used.

In the single-tissue internal validation using the FUSION method, of the 8,799 genes included in our genotype data showing significant cis-genetic expression in whole blood according to the GTEx dataset, 107 genes revealed significant association signals in the TWAS with a *p*_FDR_ less than 0.05, as detailed in Supplementary Table S2 and illustrated in Figure 2. Four candidate genes overlapped, meeting stringent criteria in both cross-tissue and single-tissue analyses, as listed in Table 1.

**TABLE 1:** Significant genes for FI in cross-tissue and single-tissue TWAS analysis.

					UTMOST_Discovery	FUSION_Replication			
Gene	Chr	BP0	BP1	Putmost	PFDR	Top GWAS ID	Z score	Pfusion	PFDR
LRPPRC	2	43996004	43996005	3.32E-05	1.03E-02	rs4953032	−5.20	1.96E-07	1.32E-04
HTT	4	3074680	3074681	1.51E-08	5.63E-05	rs362273	4.30	1.68E-05	4.04E-03
SNU13	22	41690503	41690504	1.15E-04	2.40E-02	rs1052717	−4.03	5.57E-05	1.04E-02
CCDC134	22	41800678	41800679	1.94E-04	3.15E-02	rs1052717	3.97	7.25E-05	1.22E-02

BP0, start base position; BP1, end base position; Top GWAS ID, rsID of the most significant GWAS SNP in the locus.

### 3.2 Conditional and joint analyses

**FIGURE 3:**
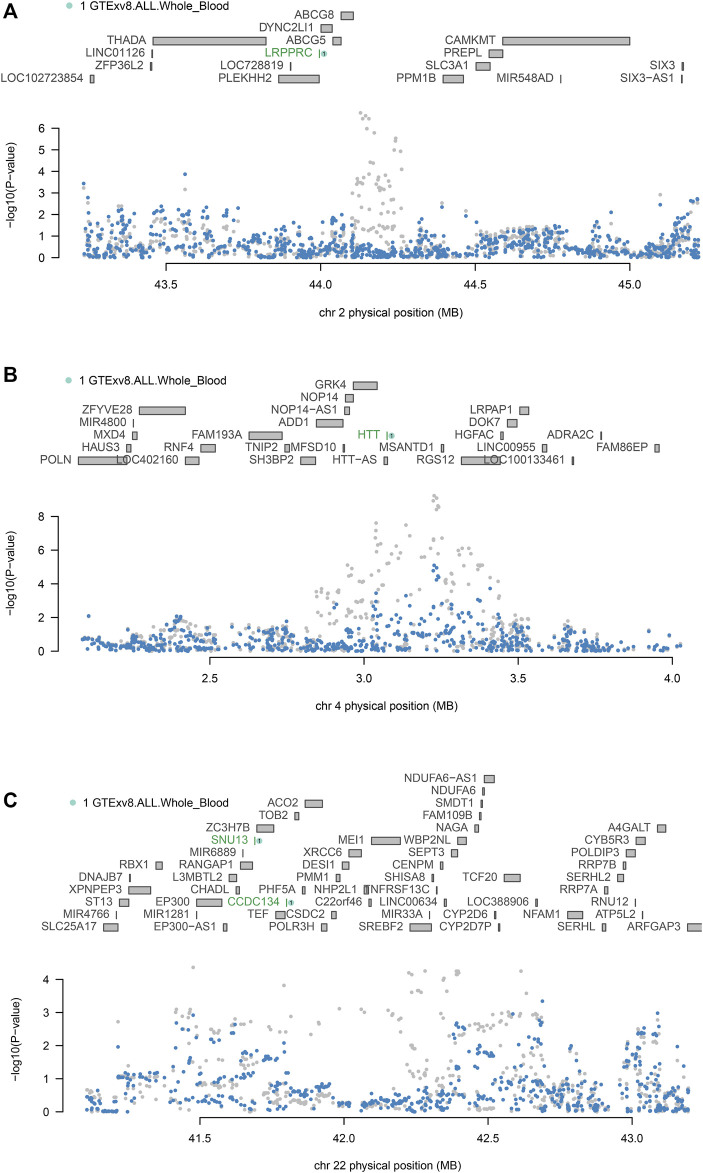
Conditional and joint analyses of FI **(A)** Chromosome 2p21 regional association plot. **(B)** Chromosome 4q16.3 regional association plot. **(C)** Chromosome 22q13.2 regional association plot. Genes independently associated with FI are highlighted in green. SNPs associated with FI before conditional analysis are highlighted in grey, and secondary SNPs associated with FI after conditional analysis are highlighted in blue.

To confirm whether genes were independently associated with phenotypes, we conducted conditional and joint analyses. Table 2 demonstrates that four distinct loci, which include essential genes such as LRPPRC (located at 2p21), HTT (at 4q16.3), and both SNU13 and CCDC134 (found at 22q13.2), serve as independent markers, each signaling different genetic information (with a conditional *p*-value of less than 0.05). It has been noted that the expression of certain genes, which are regulated genetically, can be the driving force behind some of the signals identified in GWAS. For instance, LRPPRC accounted for the majority of the signal at the 2p21 locus (Figure 3A), HTT for the majority at the 4q16.3 locus (Figure 3B), while SNU13 and CCDC134 accounted for most of the signal at the 22q13.2 locus (Figure 3C). The results for LRPPRC, HTT, SNU13, and CCDC134 remained significant after conditional analyses, indicating that these genes are independently associated with FI by themselves, not due to the locus being in linkage disequilibrium.

**TABLE 2:** Results of conditional analyses of multiple loci.

CytoBand	Gene	TWAS.Z	TWAS.P	Cond.Z	Cond.P
2p21	LRPPRC	−5.20	1.96E-07	−5.20	1.96E-07
4q16.3	HTT	4.30	1.68E-05	4.30	1.68E-05
22q13.2	SNU13	−4.03	5.57E-05	−2.40	0.02
	CCDC134	3.97	7.25E-05	2.30	0.02

Cond.P, *p*-value of the gene after conditional analysis; Cond.Z, Z-value of the gene after conditional analysis; TWAS.P, *p*-value of the gene in FUSION TWAS analysis; TWAS.Z, Z-value of the gene in FUSION TWAS analysis.

### 3.3 Statistical fine mapping

**FIGURE 4:**
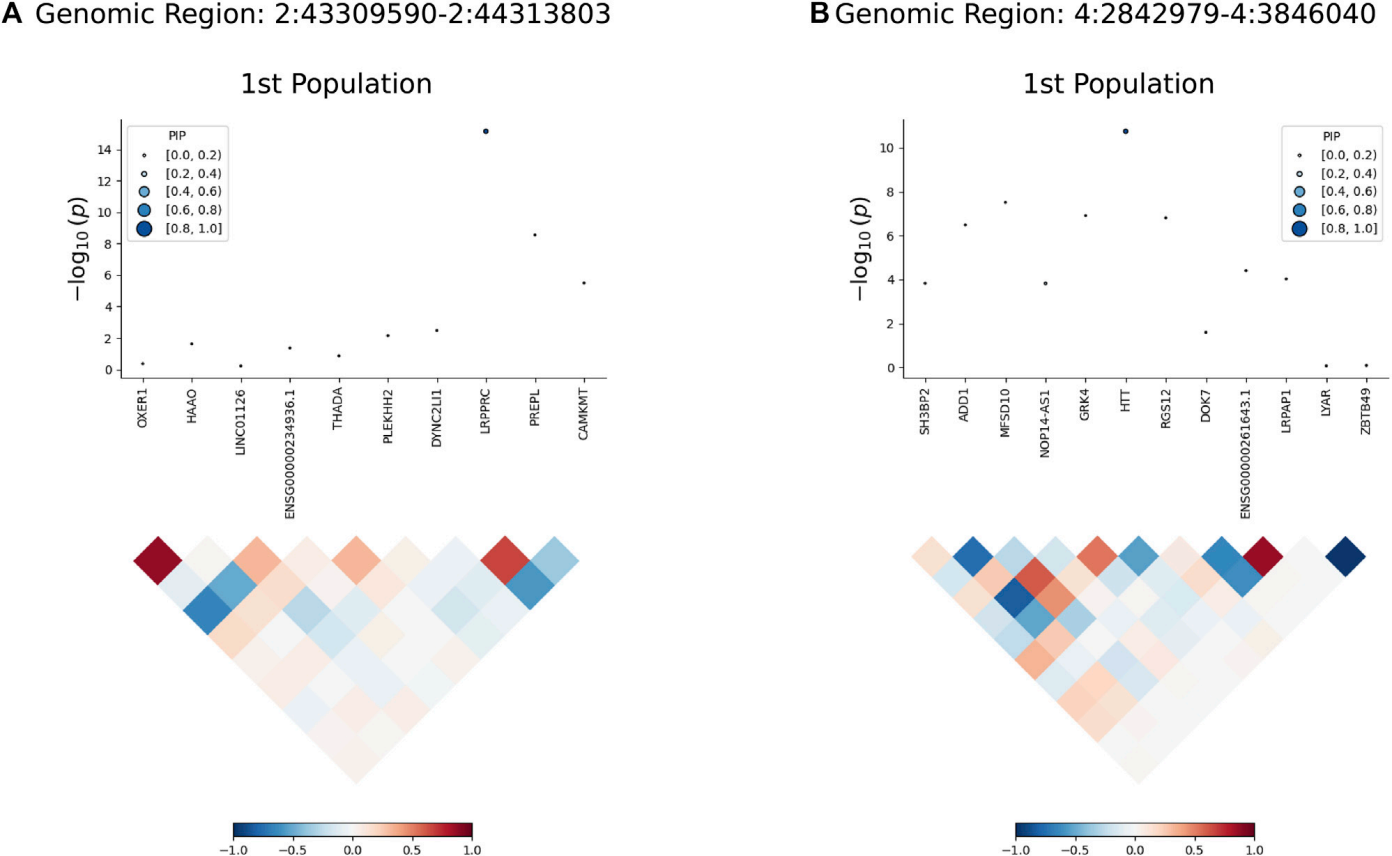
FOCUS plot for each gene in one region. **(A)** The plot contains the predicted expression correlation, TWAS summary statistics, and PIP for each gene in the genomic locus Chr2: 43996004–43996005 in the whole blood. **(B)** The plot contains the predicted expression correlation, TWAS summary statistics, and PIP for each gene in the genomic locus Chr4: 3074680–3074681 in the whole blood.

FOCUS was performed to calculate the probability estimate of causality (PIP) for each feature. We found a PIP > 0.5 for LRPPRC, HTT, suggesting that these features may be associated with FI (Table 3; Figures 4A, B). The highest probability of causality occurred in LRPPRC GTEx whole blood (PIP = 0.997).

**TABLE 3:** Statistical fine mapping results: potential causal features.

Location	Gene	SNP weight set	FOCUS PIP
Chr2:43996004–43996005	LRPPRC	GTEx whole blood	0.997
Chr4:3074680–3074681	HTT	GTEx whole blood	0.900

Chr, chromosome; GTEx, genotype tissue expression; PIP, posterior inclusion probability.

### 3.4 MAGMA analysis

**FIGURE 5:**
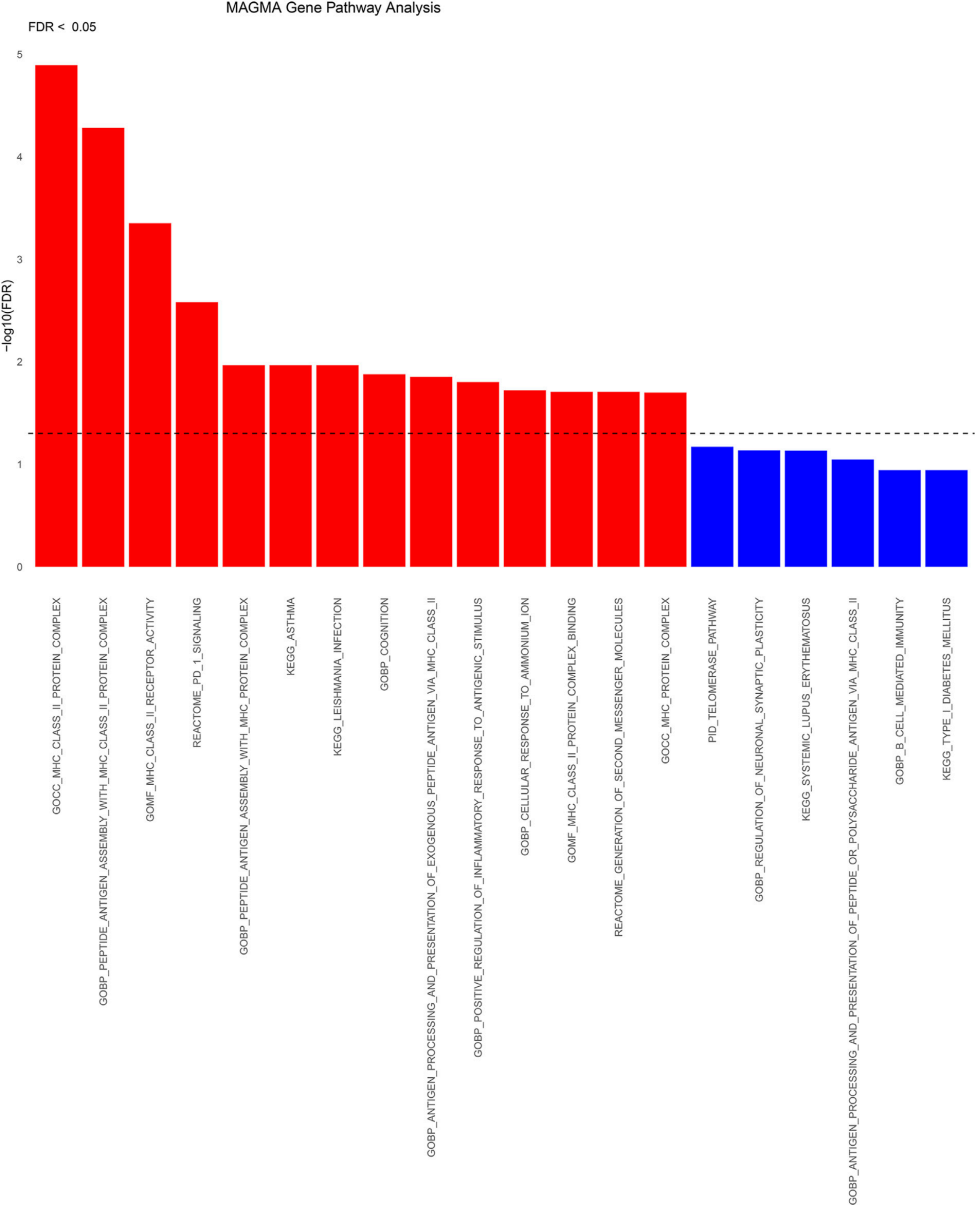
Significant types of pathways in terms of the GO and KEGG enrichment analyses through KEGG. BP, biological process; CC, cellular component; MF, molecular function; KEGG: KEGG pathways.

A total of 437 genes exhibited statistically significant signals (*p* < 0.05) following FDR correction in cross-tissue transcriptome association studies employing MAGMA (Supplementary Figure S1). Following FDR correction, these significant genes were predominantly enriched in 14 pathways primarily associated with MHC_CLASS_II, PD-1 signaling, cognition, inflammatory response to antigenic stimuli, and the production of second messenger molecules (Figure 5; Supplementary Table S3). Analyses using MAGMA focusing on tissue specificity uncovered that SNPs linked to frailty were primarily found in the cerebral frontal cortex (BA9), the broader cerebral cortex, the anterior cingulate cortex (BA24), the cerebellar hemispheres, the thalamus, and additional areas of the brain (Supplementary Figure S2).

### 3.5 Comparison of TWAS for different genetic approaches

**FIGURE 6:**
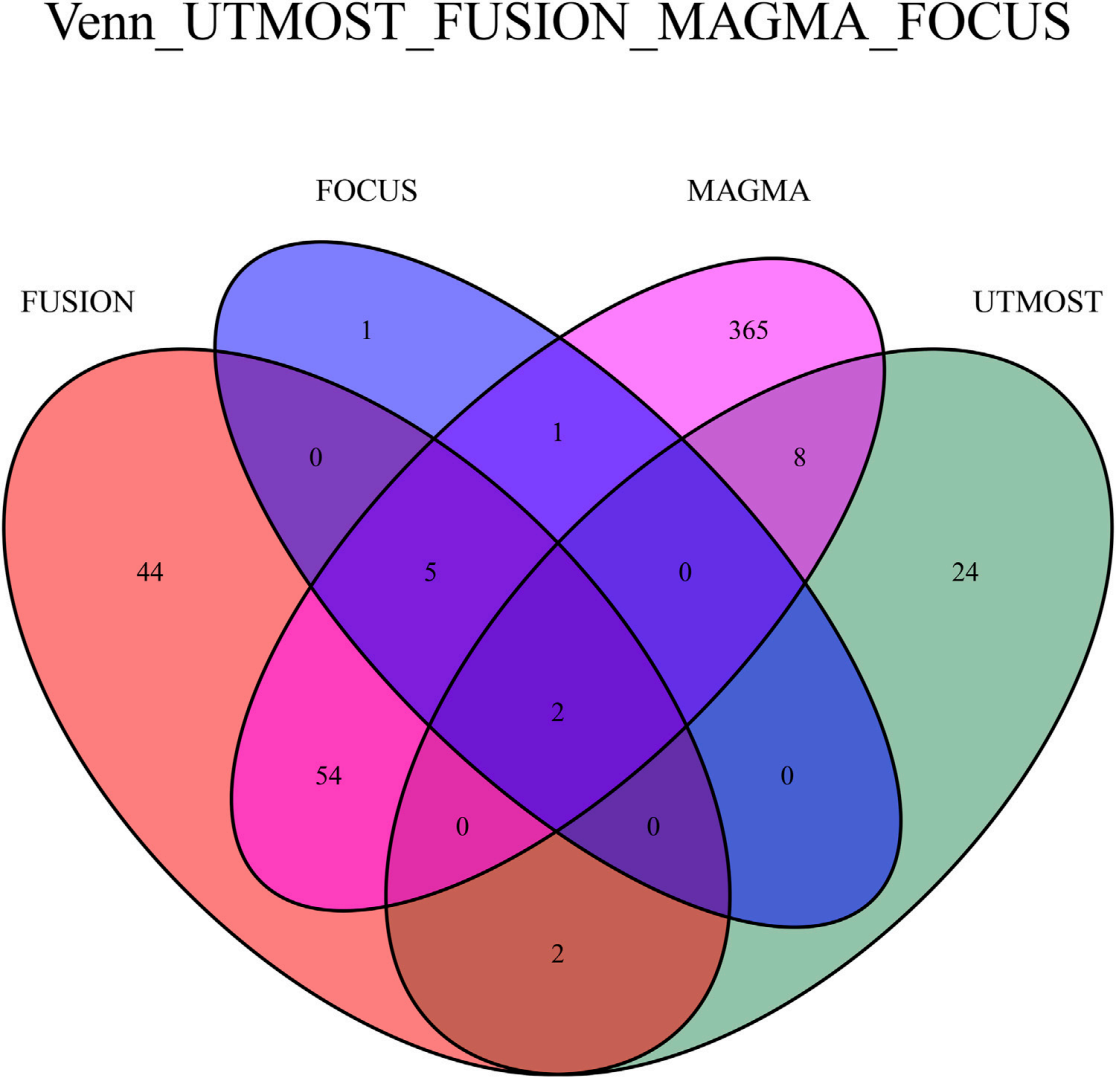
Venn plot reveals the overlap of the significant genes identified by four different methods with FDR < 0.05.

Following FDR correction, the Venn diagram demonstrates that two critical genes (LRPPRC and HTT) associated with FI were identified using four methods: UTMOST, FUSION, FOCUS, and MAGMA (Figure 6).

### 3.6 Colocation of eQTL and GWAS associations

**FIGURE 7:**
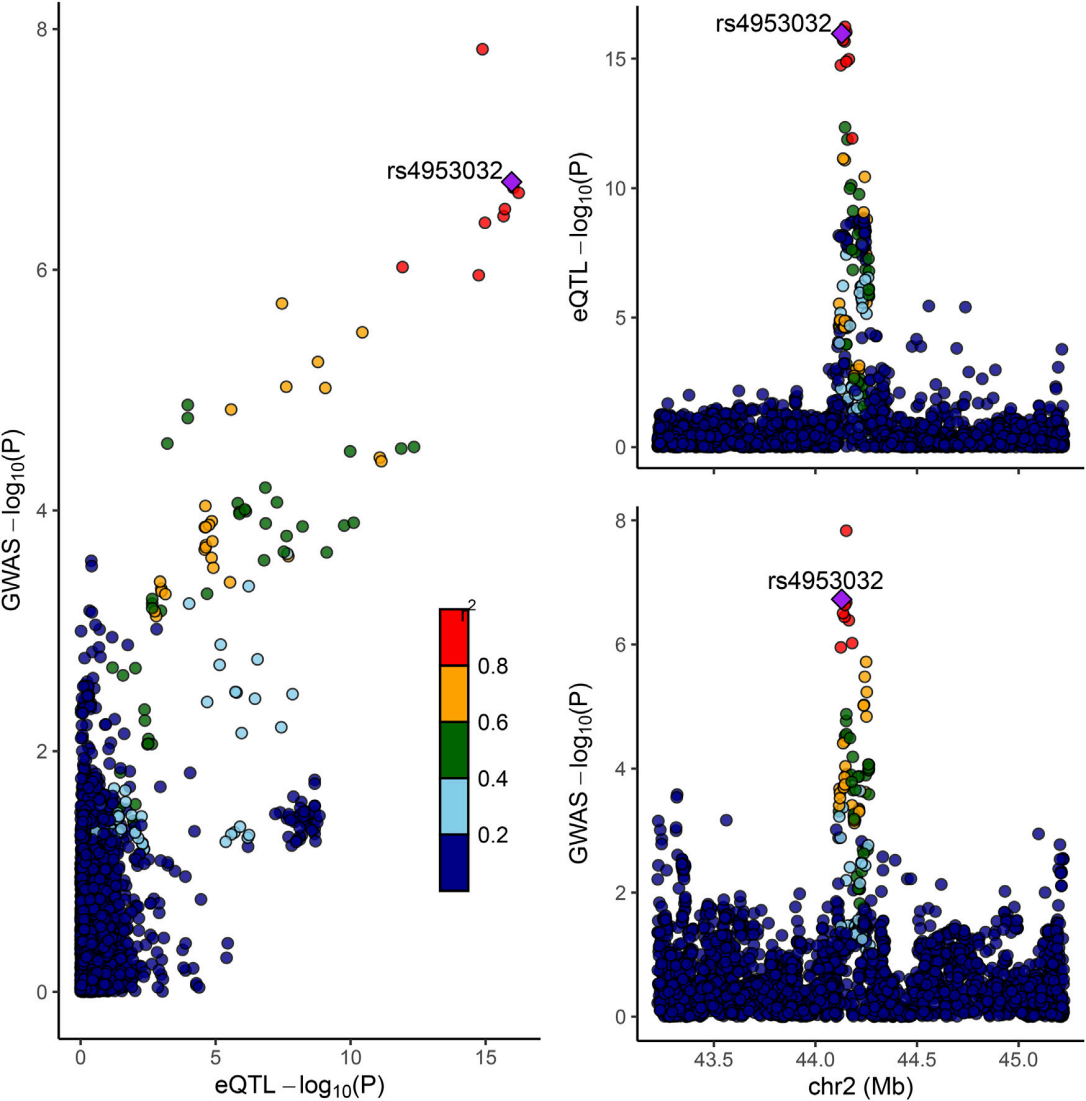
Colocation of eQTL and GWAS associations in LRPPRC. Scatterplot illustrating the overlap of GWAS and eQTL associations for LRPPRC. The *y*-axis represents GWAS *p*-values on a log10 scale for FI. The *x*-axis represents eQTL *p*-values on a −log10 scale for LRPPRC. The degree of linkage disequilibrium for all SNPs with rs4953032 is indicated by color.

Colocation analyses were then conducted to assess the likelihood that GWAS and eQTL signals are shared. Among four significant susceptibility genes in whole blood tissues (LRPPRC, HTT, SNU13, and CCDC134), it was observed that the LRPPRC gene at 2p21 may share identical GWAS and eQTL signals, evidenced by a posterior probability of PP4 (0.98) exceeding 0.75. SNPs in strong linkage disequilibrium (LD) with rs4953032 exhibited the strongest association with FI risk, with variants within 1 Mb of rs4953032 concurrently increasing the risk of FI (Figure 7).

### 3.7 Mendelian randomization

**FIGURE 8:**
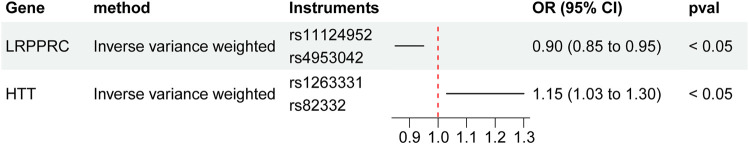
Bi-directional Mendelian Randomization (MR) analyses between LRPPRC/HTT and FI (Causal effect of LRPPRC/HTT on FI). Estimates and 95% CI were represented with square plots and error bars.

An analysis employing the two-sample Mendelian randomization approach utilized eQTL data for LRPPRC and HTT from whole blood tissues, in conjunction with GWAS data related to frailty. All SNPs utilized in the Mendelian randomization analysis were considered powerful instruments (F > 10). Thus, there is a causal relationship between the LRPPRC and HTT genes and FI. Patients carrying the LRPPRC gene are 10% less likely to develop frailty than non-carriers (OR = 0.90, 95% CI: 0.85–0.95), while patients carrying the HTT gene are 15% more likely to develop frailty than non-carriers (OR = 1.15, 95% CI: 1.03–1.30) (Figure 8).

## 4 Discussion

To date, few TWAS studies have been conducted on frailty indices, and we systematically estimated the association between genetically predicted gene expression and FI risk, utilizing the largest FI GWAS dataset available. Following FDR correction, 36 candidate genes were identified via UTMOST, and three additional validation methods (FUSION, FOCUS, MAGMA) were employed to identify more reliable genes.

The synthesis of findings from four distinct methodologies led to the identification of two genes, LRPPRC and HTT, as having a strong association with FI. This discovery offers significant new understanding of the genetic basis underpinning frailty. Research by Atkins et al. (2021) and Ye et al. (2023) identified genetic loci in the HTT gene concerning frailty, previously linked to amino acid, lipid metabolism, and BMI (Faquih et al., 2023). The difference is that the study by Atkins et al. concentrated on older adults, employing FI, whereas Ye et al. included 386,565 individuals of European descent with an average age of 57 years, using the abbreviated Fried Frailty Score (FFS) to define the frailty phenotype. Previous research has shown that the LRPPRC gene encodes a protein involved in several critical biological processes, notably in cellular energy production and metabolic regulation (Cui et al., 2019). This gene notably plays a crucial role in mitochondrial function, regulating mitochondrial RNA stability and expression, potentially influencing early, multisystem, and neurological manifestations of mitochondrial disease (Oláhová et al., 2015). Frailty is frequently linked to reduced energy metabolism, muscle hypomobility, and systemic inflammation (Barros et al., 2022); thus, the LRPPRC gene might indirectly affect these physiological processes tied to frailty through its impact on mitochondrial function. Our study demonstrated an association between this gene and FI through various TWAS methods, aligning with the findings of Willems et al. In their research, the LRPPRC gene was associated with grip strength and psychomotor deficits (Willems et al., 2017), widely regarded as indicators of muscular fitness and markers of weakness.

Our tissue enrichment analysis, conducted via MAGMA, demonstrated that genomic areas linked to SNPs associated with frailty showed elevated functional expression specifically in brain tissues, including the cerebral frontal cortex (BA9), cerebral cortex, anterior cingulate cortex (BA24), cerebellar hemispheres, thalamus, in comparison to other types of tissues. In a prospective cohort study, frailty was associated with cognitive functions related to the frontal cortex in patients with Alzheimer’s disease (Chang et al., 2022). Patients with frailty have been shown to exhibit abnormal functioning of the right prefrontal cortex during the early stages of cognitive decline (Maruya et al., 2021). As age advances, the cerebellum undergoes structural and functional changes associated with mobility and cognitive deficits, subsequently contributing to the development of frailty (Arleo et al., 2023). Patients experiencing physiological cognitive decline during aging show significant reductions in grey matter volumes in areas such as the bilateral amygdala and thalamus, the right hippocampus, the right temporo-occipital cortex, and the left cerebellar regions VI and V (Liu et al., 2020), which partially aligns with our findings.

Two genetic regions associated with frailty were identified through conditional, combined analyses, and fine mapping: 2p21 and 4q16.3. Previous research has suggested that 2p21 may be linked to Lewy body dementia (Peuralinna et al., 2015), and cognitive decline in Lewy body dementia could exacerbate frailty, affecting activities of daily living, quality of life, and independence due to impaired cognitive functioning. Research on the 4q16.3 region is limited, highlighting the importance of further investigation into the role of genes within this region concerning health and disease.

Functional enrichment analyses were also conducted using MAGMA, identifying pathways predominantly related to MHC_CLASS_II, PD-1 signaling, cognition, inflammatory response to antigenic stimuli, and the production of second messenger molecules. The MHC protein complex and PD-1 signaling are intricately linked to immune response and immunomodulation (Wilson et al., 2017; Pansarasa et al., 2019). Frailty, a complex geriatric syndrome, has etiology and pathogenesis that remain partially understood. During frailty, the immune system undergoes changes, termed “immune senescence” and “inflammation” (Tran Van Hoi et al., 2023). These phenomena are defined by imbalances in immune response related to aging, along with changes in the fundamental cellular processes. Comprehensive, untargeted LC-MS metabolomics analyses of human blood have indicated that screening for frailty markers partially correlates with cognition (Kameda et al., 2020).Inflammation is described as the overstimulation of the innate immune system due to aging, which results in a persistent, low-intensity, non-infectious state of inflammation (Soysal et al., 2016). Studies have demonstrated that older adults exhibit elevated levels of CRP, cytokines, chemokines, and abnormal leukocyte distribution, reflecting a dysregulated inflammatory state associated with aging (Marcos-Pérez et al., 2020; Minhas et al., 2021). Chronic inflammation is regarded as a key factor in the development of frailty, with intracellular second messengers like cyclic adenosine monophosphate (cAMP), calcium ions (Ca^2+^), and various phosphorylated proteins playing vital roles in the regulation of immune cells, production of inflammatory factors, and other cellular processes linked to inflammation (Langmann et al., 2017; López-Otín et al., 2023).

As research on aging progresses, identifying biological pathways and potential therapeutic targets associated with resilience becomes increasingly important, instead of focusing solely on disease risk factors (Montine et al., 2019; Vetrano et al., 2021). This paradigm shift is crucial for understanding characteristics like frailty, which reflect an individual’s overall functional status. Given this context, our study reveals novel and significant findings. Utilizing two-sample Mendelian randomization analyses, we investigated the causal relationship between specific genes and the development of frailty and discovered that HTT may elevate the risk of frailty, while LRPPRC could serve as a protective factor against its development. Furthermore, through co-localization analysis, a shared SNP between LRPPRC and FI was identified, implying LRPPRC’s central role in the development of frailty, suggesting that interventions targeting this gene or its pathways could potentially prevent or ameliorate the condition of frailty.

There are some limitations to our study. First, our data source was the frailty index, and exploring additional assessment methods for frailty, like the Fried frailty phenotype and the Edmonton Frailty Scale, could facilitate the identification of genes of interest across various dimensions, including physical performance, cognitive, and psychosocial aspects. Second, since our data predominantly come from European sources, this restricts the applicability of our findings to other ethnic groups. Therefore, it’s critical to perform similar studies among varied ethnic populations to enhance the universality of our results. Lastly, environmental factors, such as diet, physical activity, and stress, impact frailty to different extents, and employing Mendelian randomization to examine the effects of various environmental factors on frailty could aid in identifying modifiable factors that mitigate frailty’s severity or lower its risk.

## 5 Conclusion

In conclusion, this study identified two novel susceptibility genes associated with FI risk through four TWAS methods, with Mendelian randomization revealing that HTT may increase frailty risk, while LRPPRC could serve as a protective factor against its onset. Additionally, co-localization analysis identified a shared SNP between LRPPRC and FI. Tissue enrichment analysis indicated that genomic regions linked to frailty-associated SNPs were predominantly enriched in brain tissue. Conditional, combined analyses, and fine mapping pinpointed two genetic regions associated with frailty: 2p21 and 4q16.3. Functional enrichment analyses uncovered that frailty-associated pathways primarily involve the MHC complex, PD-1 signaling, cognition, inflammatory responses to antigenic stimuli, and the production of second messenger molecules. This discovery offers valuable new insights into the genetic foundations of FI and emphasizes the importance of investigating these genes’ roles in frailty development. Not only does this deepen our understanding of frailty’s biological basis, but it also promises new targets for developing preventive and therapeutic strategies against frailty.

## Data Availability

Publicly available datasets were analyzed in this study. This data can be found here: GWAS summary statistics for FI are available for download from the GWAS Catalog (study accession code GCST90020053).

## References

[B1] Arleo A. Bareš M. Bernard J. A. Bogoian H. R. Bruchhage M. Bryant P. (2023). Consensus paper: cerebellum and ageing. Cerebellum 23, 802–832. 10.1007/s12311-023-01577-7 37428408 PMC10776824

[B2] Atkins J. L. Jylhävä J. Pedersen N. L. Magnusson P. K. Lu Y. Wang Y. (2021). A genome-wide association study of the frailty index highlights brain pathways in ageing. Aging Cell 20 (9), e13459. 10.1111/acel.13459 34431594 PMC8441299

[B3] Barros D. Marques E. A. Magalhães J. Carvalho J. (2022). Energy metabolism and frailty: the potential role of exercise-induced myokines - a narrative review. Ageing Res. Rev. 82, 82101780. 10.1016/j.arr.2022.101780 36334911

[B4] Burgess S. Small D. S. Thompson S. G. (2017). A review of instrumental variable estimators for Mendelian randomization. Stat. Methods Med. Res. 26 (5), 2333–2355. 10.1177/0962280215597579 26282889 PMC5642006

[B5] Byrne E. M. Zhu Z. Qi T. Skene N. G. Bryois J. Pardinas A. F. (2021). Conditional GWAS analysis to identify disorder-specific SNPs for psychiatric disorders. Mol. Psychiatry 26 (6), 2070–2081. 10.1038/s41380-020-0705-9 32398722 PMC7657979

[B6] Cesari M. Gambassi G. van Kan G. A. Vellas B. (2014). The frailty phenotype and the frailty index: different instruments for different purposes. Age Ageing 43 (1), 10–12. 10.1093/ageing/aft160 24132852

[B7] Chang Y. S. Wang C. J. Wu C. H. Wu Y. H. Lee H. N. (2022). Frailty is associated with frontal cortex-related cognitive function in patients with alzheimer disease. J. Geriatr. Psychiatry Neurol. 35 (4), 544–549. 10.1177/08919887211016062 33977812

[B8] Cui J. Wang L. Ren X. Zhang Y. Zhang H. (2019). LRPPRC: a multifunctional protein involved in energy metabolism and human disease. Front. Physiol. 10595, 595. 10.3389/fphys.2019.00595 PMC6543908 31178748

[B9] de Leeuw C. A. Mooij J. M. Heskes T. Posthuma D. (2015). MAGMA: generalized gene-set analysis of GWAS data. PLoS Comput. Biol. 11 (4), e1004219. 10.1371/journal.pcbi.1004219 25885710 PMC4401657

[B10] Deng Y. Pan W. (2018). Improved use of small reference panels for conditional and joint analysis with GWAS summary statistics. Genetics 209 (2), 401–408. 10.1534/genetics.118.300813 29674520 PMC5972416

[B11] Dent E. Martin F. C. Bergman H. Woo J. Romero-Ortuno R. Walston J. D. (2019). Management of frailty: opportunities, challenges, and future directions. Lancet 394 (10206), 1376–1386. 10.1016/S0140-6736(19)31785-4 31609229

[B12] Faquih T. O. Aziz N. A. Gardiner S. L. Li-Gao R. de Mutsert R. Milaneschi Y. (2023). Normal range CAG repeat size variations in the HTT gene are associated with an adverse lipoprotein profile partially mediated by body mass index. Hum. Mol. Genet. 32 (10), 1741–1752. 10.1093/hmg/ddad020 36715614 PMC10448954

[B13] Ferrucci L. Fabbri E. (2018). Inflammageing: chronic inflammation in ageing, cardiovascular disease, and frailty. Nat. Rev. Cardiol. 15 (9), 505–522. 10.1038/s41569-018-0064-2 30065258 PMC6146930

[B14] Gusev A. Ko A. Shi H. Bhatia G. Chung W. Penninx B. W. (2016). Integrative approaches for large-scale transcriptome-wide association studies. Nat. Genet. 48 (3), 245–252. 10.1038/ng.3506 26854917 PMC4767558

[B15] Hoogendijk E. O. Afilalo J. Ensrud K. E. Kowal P. Onder G. Fried L. P. (2019). Frailty: implications for clinical practice and public health. Lancet 394 (10206), 1365–1375. 10.1016/S0140-6736(19)31786-6 31609228

[B16] Hu Y. Li M. Lu Q. Weng H. Wang J. Zekavat S. M. (2019). A statistical framework for cross-tissue transcriptome-wide association analysis. Nat. Genet. 51 (3), 568–576. 10.1038/s41588-019-0345-7 30804563 PMC6788740

[B17] Kameda M. Teruya T. Yanagida M. Kondoh H. (2020). Frailty markers comprise blood metabolites involved in antioxidation, cognition, and mobility. Proc. Natl. Acad. Sci. U.S.A. 117 (17), 9483–9489. 10.1073/pnas.1920795117 32295884 PMC7196897

[B18] Kojima G. Iliffe S. Walters K. (2018). Frailty index as a predictor of mortality: a systematic review and meta-analysis. Age Ageing 47 (2), 193–200. 10.1093/ageing/afx162 29040347

[B19] Langmann G. A. Perera S. Ferchak M. A. Nace D. A. Resnick N. M. Greenspan S. L. (2017). Inflammatory markers and frailty in long-term care residents. J. Am. Geriatr. Soc. 65 (8), 1777–1783. 10.1111/jgs.14876 28323342 PMC5555807

[B20] Liu L. K. Chou K. H. Hsu C. H. Peng L. N. Lee W. J. Chen W. T. (2020). Cerebellar-limbic neurocircuit is the novel biosignature of physio-cognitive decline syndrome. Aging 12 (24), 25319–25336. 10.18632/aging.104135 33234736 PMC7803525

[B21] López-Otín C. Blasco M. A. Partridge L. Serrano M. Kroemer G. (2023). Hallmarks of aging: an expanding universe. Cell 186 (2), 243–278. 10.1016/j.cell.2022.11.001 36599349

[B22] Lu Z. Gopalan S. Yuan D. Conti D. V. Pasaniuc B. Gusev A. (2022). Multi-ancestry fine-mapping improves precision to identify causal genes in transcriptome-wide association studies. Am. J. Hum. Genet. 109 (8), 1388–1404. 10.1016/j.ajhg.2022.07.002 35931050 PMC9388396

[B23] Mai J. Lu M. Gao Q. Zeng J. Xiao J. (2023). Transcriptome-wide association studies: recent advances in methods, applications and available databases. Commun. Biol. 6 (1), 899. 10.1038/s42003-023-05279-y 37658226 PMC10474133

[B24] Mancuso N. Freund M. K. Johnson R. Shi H. Kichaev G. Gusev A. (2019). Probabilistic fine-mapping of transcriptome-wide association studies. Nat. Genet. 51 (4), 675–682. 10.1038/s41588-019-0367-1 30926970 PMC6619422

[B25] Marcos-Pérez D. Sánchez-Flores M. Proietti S. Bonassi S. Costa S. Teixeira J. P. (2020). Association of inflammatory mediators with frailty status in older adults: results from a systematic review and meta-analysis. Geroscience 42 (6), 1451–1473. 10.1007/s11357-020-00247-4 32803650 PMC7732924

[B26] Maruya K. Arai T. Fujita H. (2021). Brain activity in the prefrontal cortex during cognitive tasks and dual tasks in community-dwelling elderly people with pre-frailty: a pilot study for early detection of cognitive decline. Healthc. Basel, Switz. 9 (10), 1250. 10.3390/healthcare9101250 PMC8536000 34682930

[B27] McIsaac D. I. Taljaard M. Bryson G. L. Beaulé P. E. Gagné S. Hamilton G. (2020). Frailty as a predictor of death or new disability after surgery: a prospective cohort study. Ann. Surg. 271 (2), 283–289. 10.1097/SLA.0000000000002967 30048320

[B28] Minhas P. S. Latif-Hernandez A. McReynolds M. R. Durairaj A. S. Wang Q. Rubin A. (2021). Restoring metabolism of myeloid cells reverses cognitive decline in ageing. Nature 590 (7844), 122–128. 10.1038/s41586-020-03160-0 33473210 PMC8274816

[B29] Montine T. J. Cholerton B. A. Corrada M. M. Edland S. D. Flanagan M. E. Hemmy L. S. (2019). Concepts for brain aging: resistance, resilience, reserve, and compensation. Alzheimer's Res. Ther. 11 (1), 22. 10.1186/s13195-019-0479-y 30857563 PMC6410486

[B30] NguyenT. N.CummingR. G.HilmerS. N. (2015). A review of frailty in developing countries. J. Nutr. Health Aging 19 (9), 941–946. 10.1007/s12603-015-0503-2 26482697

[B31] O'Caoimh R. Sezgin D. O'Donovan M. R. Molloy D. W. Clegg A. Rockwood K. (2021). Prevalence of frailty in 62 countries across the world: a systematic review and meta-analysis of population-level studies. Age Ageing 50 (1), 96–104. 10.1093/ageing/afaa219 33068107

[B32] Oláhová M. Hardy S. A. Hall J. Yarham J. W. Haack T. B. Wilson W. C. (2015). LRPPRC mutations cause early-onset multisystem mitochondrial disease outside of the French-Canadian population. Brain 138 (Pt 12), 3503–3519. 10.1093/brain/awv291 26510951 PMC4655343

[B33] Pansarasa O. Pistono C. Davin A. Bordoni M. Mimmi M. C. Guaita A. (2019). Altered immune system in frailty: genetics and diet may influence inflammation. Ageing Res. Rev. 54100935, 100935. 10.1016/j.arr.2019.100935 31326616

[B34] Peuralinna T. Myllykangas L. Oinas M. Nalls M. A. Keage H. A. Isoviita V. M. (2015). Genome-wide association study of neocortical Lewy-related pathology. Ann. Clin. Transl. Neurol. 2 (9), 920–931. 10.1002/acn3.231 26401513 PMC4574809

[B35] Rodriguez-Fontenla C. Carracedo A. (2021). UTMOST, a single and cross-tissue TWAS (transcriptome wide association study), reveals new ASD (autism spectrum disorder) associated genes. Transl. Psychiatry 11 (1), 256. 10.1038/s41398-021-01378-8 33931583 PMC8087708

[B36] Sey N. Pratt B. M. Won H. (2023). Annotating genetic variants to target genes using H-MAGMA. Nat. Protoc. 18 (1), 22–35. 10.1038/s41596-022-00745-z 36289406 PMC10026181

[B37] Shi S. M. Olivieri-Mui B. McCarthy E. P. Kim D. H. (2021). Changes in a frailty index and association with mortality. J. Am. Geriatr. Soc. 69 (4), 1057–1062. 10.1111/jgs.17002 33377190 PMC8071066

[B38] Soysal P. Stubbs B. Lucato P. Luchini C. Solmi M. Peluso R. (2016). Inflammation and frailty in the elderly: a systematic review and meta-analysis. Ageing Res. Rev. 31, 311–318. 10.1016/j.arr.2016.08.006 27592340

[B39] Tran Van Hoi E. De Glas N. A. Portielje J. Van Heemst D. Van Den Bos F. Jochems S. P. (2023). Biomarkers of the ageing immune system and their association with frailty - a systematic review. Exp. Gerontol. 176112163, 112163. 10.1016/j.exger.2023.112163 37028607

[B40] Vetrano D. L. Triolo F. Maggi S. Malley R. Jackson T. A. Poscia A. (2021). Fostering healthy aging: the interdependency of infections, immunity and frailty. Ageing Res. Rev. 69, 69101351. 10.1016/j.arr.2021.101351 PMC9588151 33971332

[B41] Wainberg M. Sinnott-Armstrong N. Mancuso N. Barbeira A. N. Knowles D. A. Golan D. (2019). Opportunities and challenges for transcriptome-wide association studies. Nat. Genet. 51 (4), 592–599. 10.1038/s41588-019-0385-z 30926968 PMC6777347

[B42] Willems S. M. Wright D. J. Day F. R. Trajanoska K. Joshi P. K. Morris J. A. (2017). Large-scale GWAS identifies multiple loci for hand grip strength providing biological insights into muscular fitness. Nat. Commun. 8, 816015. 10.1038/ncomms16015 PMC5510175 29313844

[B43] Wilson D. Jackson T. Sapey E. Lord J. M. (2017). Frailty and sarcopenia: the potential role of an aged immune system. Ageing Res. Rev. 361-10, 1–10. 10.1016/j.arr.2017.01.006 28223244

[B44] Ye Y. Noche R. B. Szejko N. Both C. P. Acosta J. N. Leasure A. C. (2023). A genome-wide association study of frailty identifies significant genetic correlation with neuropsychiatric, cardiovascular, and inflammation pathways. Geroscience 45 (4), 2511–2523. 10.1007/s11357-023-00771-z 36928559 PMC10651618

