# Supplementary material for: A cross-tissue transcriptome-wide association study identifies new susceptibility genes for frailty
Source: Front Genet. 2024 Jul 12;15:1404456. doi: 10.3389/fgene.2024.1404456 (PMC11272547; doi:10.3389/fgene.2024.1404456)
Supplement: Supplementary file 1 [file Table1.DOCX]

**Supplemental Table S1.** TWAS summary statistics in cross-tissue test using 44 GTEx tissues (P_FDR_<0.05)

| **symble** | **test_score** | **p_value** | **P_FDR_** |
| --- | --- | --- | --- |
| HTT | 17.69694558 | 1.51E-08 | 5.63E-05 |
| MFSD10 | 13.94760059 | 5.74E-07 | 0.000599133 |
| HAUS3 | 13.98262027 | 6.45E-07 | 0.000599133 |
| RP11-357G3.2 | 14.56928338 | 4.80E-07 | 0.000599133 |
| SLC39A8 | 12.49422994 | 1.92E-06 | 0.00118859 |
| MSANTD1 | 12.19160575 | 1.88E-06 | 0.00118859 |
| CISD2 | 10.47335605 | 6.13E-06 | 0.002528608 |
| CCSER1 | 12.02724558 | 6.11E-06 | 0.002528608 |
| RP11-10L12.4 | 10.88416236 | 4.81E-06 | 0.002528608 |
| UBE2D3 | 10.0257924 | 1.63E-05 | 0.006037867 |
| LRPPRC | 8.545676388 | 3.32E-05 | 0.010282332 |
| LRRC37A15P | 8.906632688 | 3.25E-05 | 0.010282332 |
| ZNF396 | 9.688622525 | 4.53E-05 | 0.01294144 |
| NOP14 | 10.03246676 | 5.60E-05 | 0.01484362 |
| SNU13 | 8.855519865 | 0.00011469 | 0.024036219 |
| OTOF | 8.872366928 | 0.000117959 | 0.024036219 |
| ZAR1 | 9.134418297 | 0.000110343 | 0.024036219 |
| AC007879.5 | 9.057929262 | 0.000122964 | 0.024036219 |
| KRT8P46 | 7.806667848 | 0.000119053 | 0.024036219 |
| UBA6 | 7.698357243 | 0.000222719 | 0.031517484 |
| CCDC134 | 7.760281473 | 0.000194103 | 0.031517484 |
| EP300 | 8.099638101 | 0.000181092 | 0.031517484 |
| GTDC1 | 8.383397646 | 0.000205591 | 0.031517484 |
| SPHKAP | 8.296876461 | 0.000227679 | 0.031517484 |
| RP5-821D11.7 | 8.168726373 | 0.000206013 | 0.031517484 |
| RP11-763F8.1 | 3.690956876 | 0.000223412 | 0.031517484 |
| NOP14-AS1 | 7.726748147 | 0.000229125 | 0.031517484 |
| RTP5 | 7.757153242 | 0.000250509 | 0.033228291 |
| TMPRSS11CP | 27.62821218 | 0.000265867 | 0.034049308 |
| RN7SKP292 | 8.136654093 | 0.000305671 | 0.03784202 |
| ITGB6 | 7.376580771 | 0.00034536 | 0.040527683 |
| WBP2NL | 6.705040147 | 0.000349188 | 0.040527683 |
| CAD | 7.001633925 | 0.000383382 | 0.041878822 |
| MANBA | 8.03266372 | 0.000380242 | 0.041878822 |
| DNAJC5G | 7.790847722 | 0.000395159 | 0.041932065 |
| ARHGAP15 | 6.944160961 | 0.000483608 | 0.049892209 |

**Supplementary Table S2.** TWAS summary statistics in single-tissue test using whole blood eQTL from GTEx

| **ID** | **CHR** | **BEST.GWAS.ID** | **BEST.GWAS.Z** | **EQTL.ID** | **EQTL.Z** | **EQTL.GWAS.Z** | **NSNP** | **TWAS.Z** | **TWAS.P** | **FDR** |
| --- | --- | --- | --- | --- | --- | --- | --- | --- | --- | --- |
| HLA-DQB2 | 6 | rs660895 | 9.68 | rs1063355 | -21 | -8.3529 | 260 | 8.54944 | 1.24E-17 | 1.09E-13 |
| HLA-DQA1 | 6 | rs660895 | 9.68 | rs1063355 | 15.32 | -8.3529 | 220 | -8.30371 | 1.01E-16 | 4.42E-13 |
| HLA-DQB1 | 6 | rs660895 | 9.68 | rs1063355 | 21.4 | -8.3529 | 227 | -7.58297 | 3.38E-14 | 9.86E-11 |
| HLA-DQA2 | 6 | rs660895 | 9.68 | rs28366298 | 19.09 | 2.6667 | 260 | 7.31216 | 2.63E-13 | 5.75E-10 |
| PRRC2A | 6 | rs707939 | 6.94 | rs2075800 | 7.41 | 6.5833 | 283 | 7.16956 | 7.52E-13 | 1.32E-09 |
| BAG6 | 6 | rs707939 | 6.94 | rs494620 | 4.24 | 6.1471 | 242 | 6.30834 | 2.82E-10 | 4.11E-07 |
| ABHD16A | 6 | rs707939 | 6.94 | rs2515919 | 3.62 | 6.1471 | 242 | 5.88129 | 4.07E-09 | 5.09E-06 |
| HLA-DRB1 | 6 | rs660895 | 9.68 | rs28366298 | -10.56 | 2.6667 | 239 | -5.67823 | 1.36E-08 | 1.49E-05 |
| RBM6 | 3 | rs2013208 | -5.67 | rs7613875 | 18.94 | -5.4545 | 323 | -5.53399 | 3.13E-08 | 3.04E-05 |
| CHP1 | 15 | rs2176998 | 5.24 | rs2928155 | -5.57 | 4.9412 | 271 | -5.50388 | 3.72E-08 | 3.26E-05 |
| SLC44A4 | 6 | rs707939 | 6.94 | rs605203 | 4.88 | -5.2353 | 226 | -5.43606 | 5.45E-08 | 3.97E-05 |
| DDAH2 | 6 | rs707939 | 6.94 | rs3130287 | 5.71 | -5.1087 | 242 | -5.44208 | 5.27E-08 | 3.97E-05 |
| LRPPRC | 2 | rs4953032 | -5.206 | rs7568481 | 7.8 | -5.1471 | 504 | -5.203279 | 1.96E-07 | 0.000131953 |
| UBA7 | 3 | rs2013208 | -5.67 | rs7372966 | -14.39 | 5.3636 | 317 | -5.12984 | 2.90E-07 | 0.000181291 |
| TMOD2 | 15 | rs2303443 | -5.42 | rs7174497 | 12.91 | -4.9706 | 421 | -4.97059 | 6.68E-07 | 0.000365396 |
| CRLF3 | 17 | rs8075341 | -4.82 | rs11656419 | -6.15 | -4.7843 | 253 | 4.97564 | 6.50E-07 | 0.000365396 |
| RPE | 2 | rs10932334 | 5.121 | rs12474078 | -3.49 | 4.9394 | 258 | -4.939394 | 7.84E-07 | 0.000403622 |
| MMAB | 12 | rs3825393 | -5.06 | rs12314392 | -5.44 | 4.8182 | 440 | -4.82 | 1.45E-06 | 0.000691408 |
| CTD-2349P21.10 | 17 | rs8075341 | -4.82 | rs11652631 | 4.4 | -4.7647 | 244 | -4.80092 | 1.58E-06 | 0.000691408 |
| NUDT18 | 8 | rs2279355 | -5.42 | rs2279355 | 15.85 | -5.4242 | 428 | -4.8097 | 1.51E-06 | 0.000691408 |
| MST1R | 3 | rs2013208 | -5.67 | rs7430334 | -5.09 | 4.1818 | 327 | -4.70785 | 2.50E-06 | 0.001041905 |
| AGER | 6 | rs660895 | 9.68 | rs3130286 | -3.83 | -4.4359 | 170 | 4.65505 | 3.24E-06 | 0.001288931 |
| CYP21A1P | 6 | rs707939 | 6.94 | rs1150753 | -9.62 | -0.6531 | 197 | 4.63213 | 3.62E-06 | 0.001377489 |
| GMPPB | 3 | rs2013208 | -5.67 | rs11720705 | 7.15 | 4.7674 | 317 | 4.61983 | 3.84E-06 | 0.00140032 |
| LANCL1 | 2 | rs10932334 | 5.121 | rs1044708 | -5.36 | -4.6061 | 341 | 4.606061 | 4.10E-06 | 0.001427249 |
| SUZ12P1 | 17 | rs8075341 | -4.82 | rs9893922 | -7.01 | -4.6471 | 263 | 4.59942 | 4.24E-06 | 0.001427249 |
| XXcos-LUCA16.1 | 3 | rs2013208 | -5.67 | rs17657664 | -4 | 4.1515 | 313 | -4.56037 | 5.11E-06 | 0.001656397 |
| MVK | 12 | rs3825393 | -5.06 | rs10161126 | 10 | -4.5455 | 441 | -4.55 | 5.48E-06 | 0.001712891 |
| XXbac-BPG252P9.10 | 6 | rs2233981 | 4.69 | rs2074478 | -4.48 | -4.5 | 77 | 4.5 | 6.80E-06 | 0.002052193 |
| HLA-DQB1-AS1 | 6 | rs660895 | 9.68 | rs1063355 | 13.31 | -8.3529 | 220 | -4.48645 | 7.24E-06 | 0.002112149 |
| RCOR1 | 14 | rs4906230 | 4.6 | rs4906255 | 8.26 | 4.4118 | 306 | 4.3874 | 1.15E-05 | 0.00324671 |
| PABPC4 | 1 | rs12037222 | 4.675 | rs4660293 | -6.24 | 4.5897 | 385 | -4.37152 | 1.23E-05 | 0.00336405 |
| ARFRP1 | 20 | rs2315008 | -4.31 | rs2315007 | 6.98 | -4.2857 | 424 | -4.31417 | 1.60E-05 | 0.004044843 |
| MON1A | 3 | rs2013208 | -5.67 | rs11130237 | -5.61 | 2.4667 | 322 | -4.29938 | 1.71E-05 | 0.004044843 |
| ACSF3 | 16 | rs555191 | 3.66 | rs7188200 | 6.11 | -1.1463 | 434 | -4.30231 | 1.69E-05 | 0.004044843 |
| HTT | 4 | rs362273 | -6.19 | rs2798289 | -7.59 | -4.303 | 407 | 4.30303 | 1.68E-05 | 0.004044843 |
| DDR1 | 6 | rs2523589 | -4.88 | rs28732100 | 4.67 | 1.892 | 93 | 4.30567 | 1.66E-05 | 0.004044843 |
| LIME1 | 20 | rs2315008 | -4.31 | rs4809330 | 5.28 | -4.2857 | 425 | -4.285714 | 1.82E-05 | 0.004191747 |
| PBX2 | 6 | rs660895 | 9.68 | rs6902493 | 3.96 | 5.75 | 170 | 4.26579 | 1.99E-05 | 0.004465764 |
| ANKK1 | 11 | rs7108081 | 5.53 | rs10891549 | -9.26 | 4.4848 | 508 | -4.19484 | 2.73E-05 | 0.00597324 |
| ZBTB20 | 3 | rs6793973 | -4.5 | rs7643617 | 4.65 | -4.125 | 304 | -4.125 | 3.71E-05 | 0.007919493 |
| RPL10AP1 | 14 | rs3212028 | 3.84 | rs8014013 | 5.94 | -2.6216 | 352 | -4.1165 | 3.85E-05 | 0.008022667 |
| FAM160B2 | 8 | rs2279355 | -5.42 | rs2279355 | 5.68 | -5.4242 | 429 | -4.09026 | 4.31E-05 | 0.008572982 |
| DDX39B | 6 | rs707939 | 6.94 | rs6929796 | -10.52 | 2.9545 | 277 | -4.09163 | 4.28E-05 | 0.008572982 |
| RP11-819C21.1 | 11 | rs999985 | 4 | rs3758911 | -5.39 | 3.7143 | 466 | -4.04887 | 5.15E-05 | 0.010016178 |
| TANK | 2 | rs270916 | -3.194 | rs4664398 | -3.71 | 2.119 | 365 | -4.042563 | 5.29E-05 | 0.0100648 |
| SNU13 | 22 | rs1052717 | -4.03 | rs1052717 | 4.64 | -4.0303 | 298 | -4.0303 | 5.57E-05 | 0.010372051 |
| BMP8A | 1 | rs12037222 | 4.675 | rs4660762 | -5.1 | -3.0294 | 375 | 4.02306 | 5.74E-05 | 0.010377371 |
| LINC01184 | 5 | rs1003136 | -4.324 | rs1112956 | -7.04 | -3.6053 | 375 | 4.02029 | 5.81E-05 | 0.010377371 |
| AC007879.2 | 2 | rs1263674 | 5.429 | rs12328009 | -5.82 | 3.9429 | 457 | -3.985033 | 6.75E-05 | 0.0118152 |
| CCDC134 | 22 | rs1052717 | -4.03 | rs7285782 | 9.55 | 4 | 310 | 3.9678 | 7.25E-05 | 0.012202308 |
| HPD | 12 | rs2707068 | 4.35 | rs2596140 | -11.98 | -3.9697 | 341 | 3.97 | 7.20E-05 | 0.012202308 |
| TMEM106B | 7 | rs17165701 | 4.56 | rs6966915 | 10.6 | 3.7273 | 664 | 3.947 | 7.91E-05 | 0.012984785 |
| NEK11 | 3 | rs12639350 | 4 | rs12639350 | 7.91 | 4 | 320 | 3.93964 | 8.16E-05 | 0.012984785 |
| TMEM258 | 11 | rs102275 | -4.29 | rs968567 | 6.04 | -2.8095 | 376 | -3.94276 | 8.06E-05 | 0.012984785 |
| NUTM2A-AS1 | 10 | rs4933426 | 3.68 | rs4934297 | -7.77 | 3.4118 | 274 | -3.9316 | 8.44E-05 | 0.013190514 |
| DAG1 | 3 | rs17080528 | -5.58 | rs885592 | -3.36 | 2.4848 | 289 | -3.92421 | 8.70E-05 | 0.013358316 |
| MSH5 | 6 | rs707939 | 6.94 | rs2075789 | -4.84 | -0.1034 | 242 | 3.89879 | 9.67E-05 | 0.014591697 |
| USP37 | 2 | rs7574429 | 3.941 | rs832813 | 5.95 | 3.8529 | 350 | 3.852941 | 0.000117 | 0.0170664 |
| CNOT9 | 2 | rs7574429 | 3.941 | rs832813 | -12.36 | 3.8529 | 350 | -3.852941 | 0.000117 | 0.0170664 |
| RCC1L | 7 | rs17207196 | -3.55 | rs2267812 | 4.22 | 2.6829 | 70 | 3.8092 | 0.000139 | 0.019943082 |
| SLC22A5 | 5 | rs20541 | 4.093 | rs2631360 | -11.79 | -3.7879 | 400 | 3.75281 | 0.000175 | 0.024703226 |
| PROB1 | 5 | rs11242465 | -3.784 | rs10063949 | -6.73 | -3.4571 | 260 | 3.74876 | 0.000178 | 0.024727873 |
| ARL11 | 13 | rs9568292 | 3.74 | rs9568292 | -5.89 | 3.74359 | 375 | -3.74359 | 0.000181 | 0.02475175 |
| DNAJC18 | 5 | rs11242465 | -3.784 | rs11242465 | 7.87 | -3.7838 | 258 | -3.73446 | 0.000188 | 0.025313477 |
| CACYBP | 1 | rs2105949 | 4.028 | rs1046439 | 6.38 | 3.7222 | 433 | 3.72222 | 0.000197 | 0.025483765 |
| AMT | 3 | rs17080528 | -5.58 | rs3448 | -13.26 | 2.9211 | 288 | -3.72198 | 0.000198 | 0.025483765 |
| SLC22A4 | 5 | rs20541 | 4.093 | rs17622208 | -5.02 | -3.7273 | 392 | 3.72727 | 0.000194 | 0.025483765 |
| CUL9 | 6 | rs6924555 | 3.93 | rs6924555 | -6.3 | 3.9348 | 343 | -3.6739 | 0.000239 | 0.029051778 |
| HPCAL4 | 1 | rs12037222 | 4.675 | rs784600 | 12.4 | 2.8182 | 384 | 3.67339 | 0.000239 | 0.029051778 |
| PPP6C | 9 | rs589292 | -5.03 | rs10760394 | -6.2 | -0.9091 | 320 | 3.67747 | 0.000236 | 0.029051778 |
| RHOF | 12 | rs2707068 | 4.35 | rs921286 | -3.62 | 2.5581 | 334 | -3.68 | 0.00023 | 0.029051778 |
| GAPT | 5 | rs4700295 | -3.735 | rs1389308 | -11.64 | -3.6471 | 523 | 3.64706 | 0.000265 | 0.031341622 |
| CLN8 | 8 | rs10091778 | 4.65 | rs7846610 | -8.87 | -1.9722 | 725 | 3.64903 | 0.000263 | 0.031341622 |
| ZCCHC4 | 4 | rs759245 | 3.76 | rs6832360 | 5.26 | 3.6364 | 547 | 3.63636 | 0.000277 | 0.032324053 |
| GRPEL1 | 4 | rs3951346 | 4.3 | rs3822270 | -5.72 | 4.25 | 634 | -3.62948 | 0.000284 | 0.032651692 |
| SUMF1 | 3 | rs807785 | -3.11 | rs304078 | -7.32 | -2.6765 | 633 | 3.62467 | 0.000289 | 0.032651692 |
| HLA-DRB5 | 6 | rs660895 | 9.68 | rs9271055 | 19.04 | -4.9556 | 262 | -3.62339 | 0.000291 | 0.032651692 |
| ZNF23 | 16 | rs9934519 | 4.26 | rs13333985 | 4 | 3.3056 | 317 | 3.61558 | 3.00E-04 | 0.033235443 |
| NME7 | 1 | rs10919144 | -4.727 | rs12751062 | 6.2 | -3.3939 | 590 | -3.60403 | 0.000313 | 0.033819457 |
| SPATA24 | 5 | rs11242465 | -3.784 | rs10900862 | 5.18 | -3.2 | 261 | -3.60479 | 0.000312 | 0.033819457 |
| FMNL3 | 12 | rs2303305 | -3.72 | rs11833411 | -4.45 | -3.6 | 336 | 3.6 | 0.000318 | 0.033940683 |
| TOP2B | 3 | rs2293787 | -3.72 | rs1881708 | -4.79 | -3.5 | 470 | 3.59036 | 0.00033 | 0.034797108 |
| ZNF24 | 18 | rs2978360 | -4.27 | rs9959139 | 7.49 | -4.1515 | 405 | -3.58119 | 0.000342 | 0.035633143 |
| CDK2AP1 | 12 | rs11061244 | 4.04 | rs7315453 | 13.34 | 3.2632 | 341 | 3.57 | 0.000355 | 0.036552471 |
| VARS2 | 6 | rs2523589 | -4.88 | rs2523578 | -5.78 | -3.5833 | 93 | 3.56449 | 0.000365 | 0.037120552 |
| FADS1 | 11 | rs102275 | -4.29 | rs968567 | 5.53 | -2.8095 | 398 | -3.56159 | 0.000369 | 0.037120552 |
| CENPM | 22 | rs1052717 | -4.03 | rs17002947 | -4.11 | -3.0952 | 336 | 3.549 | 0.000387 | 0.038488909 |
| PMS2P3 | 7 | rs17148752 | -3.66 | rs17207196 | 8.78 | -3.5455 | 215 | -3.5455 | 0.000392 | 0.038548135 |
| ABO | 9 | rs8176720 | -3.83 | rs8176749 | 13.62 | -2.2941 | 565 | -3.53937 | 0.000401 | 0.038566505 |
| USP32P3 | 17 | rs12451841 | -3.97 | rs10459970 | 8.1 | 2.3878 | 176 | 3.54223 | 0.000397 | 0.038566505 |
| RTF1 | 15 | rs2176998 | 5.24 | rs9302109 | 6.28 | 3.4857 | 301 | 3.51128 | 0.000446 | 0.042428174 |
| LRRC37A17P | 17 | rs11079763 | -3.29 | rs11079750 | -6.85 | -2.9394 | 294 | 3.5078 | 0.000452 | 0.042536602 |
| AC002310.14 | 16 | rs9924308 | 3.61 | rs2285459 | 3.84 | 3.1818 | 185 | 3.50306 | 0.00046 | 0.042828936 |
| RGS12 | 4 | rs362273 | -6.19 | rs3129308 | 5.51 | -4.7647 | 412 | -3.49472 | 0.000475 | 0.04376 |
| BAG5 | 14 | rs3212028 | 3.84 | rs34026011 | 6.03 | 3.4865 | 349 | 3.4865 | 0.000489 | 0.0445805 |
| ARHGEF10 | 8 | rs10091778 | 4.65 | rs7007884 | 6.63 | 3.4722 | 676 | 3.47222 | 0.000516 | 0.04568544 |
| LSR | 19 | rs12975066 | 3.64 | rs17541474 | -4.03 | 2.2143 | 485 | -3.469171 | 0.000522 | 0.04568544 |
| ERO1A | 14 | rs802991 | -3.34 | rs3783453 | -8.09 | 0.4444 | 361 | -3.4765 | 0.000508 | 0.04568544 |
| RP11-333E1.2 | 17 | rs3760490 | 3.9 | rs9914449 | 5.49 | -3.4706 | 460 | -3.47059 | 0.000519 | 0.04568544 |
| TMCO6 | 5 | rs2337515 | -3.875 | rs2073512 | 5.65 | -3.4615 | 343 | -3.46154 | 0.000537 | 0.045863846 |
| ITPKA | 15 | rs2176998 | 5.24 | rs1617832 | 5.39 | 4.2 | 327 | 3.45737 | 0.000545 | 0.045863846 |
| TTC12 | 11 | rs7108081 | 5.53 | rs723077 | -11.89 | -0.8485 | 518 | 3.45769 | 0.000545 | 0.045863846 |
| ARHGAP1 | 11 | rs7940240 | -3.59 | rs10501319 | -3.5 | -3.2564 | 236 | 3.46243 | 0.000535 | 0.045863846 |
| MST1 | 3 | rs2013208 | -5.67 | rs13100791 | 4.92 | -3.7246 | 316 | -3.44814 | 0.000564 | 0.046567245 |
| SMG1P5 | 16 | rs9924308 | 3.61 | rs6565176 | -8.3 | 3.3636 | 209 | -3.4509 | 0.000559 | 0.046567245 |
| C5orf34 | 5 | rs7715782 | -3.515 | rs6878770 | 7.83 | -3.4242 | 256 | -3.43968 | 0.000582 | 0.047604336 |

**Supplementary Table S3. Gene-set analysis of significant genes identified by MAGMA**

| **FULL_NAME** | **NGENES** | **BETA** | **BETA_STD** | **SE** | **P** | **FDR** | **logFDR** |
| --- | --- | --- | --- | --- | --- | --- | --- |
| GOCC_MHC_CLASS_II_PROTEIN_COMPLEX | 15 | 1.671 | 0.047174 | 0.27632 | 7.51E-10 | 1.28E-05 | 4.893764648 |
| GOBP_PEPTIDE_ANTIGEN_ASSEMBLY_WITH_MHC_CLASS_II_PROTEIN_COMPLEX | 14 | 1.6534 | 0.045094 | 0.29012 | 6.12E-09 | 5.21E-05 | 4.283313201 |
| GOMF_MHC_CLASS_II_RECEPTOR_ACTIVITY | 9 | 2.007 | 0.043894 | 0.38252 | 7.84E-08 | 0.000444226 | 3.35239575 |
| REACTOME_PD_1_SIGNALING | 21 | 1.0375 | 0.03465 | 0.21381 | 6.15E-07 | 0.002615915 | 2.582376299 |
| GOBP_PEPTIDE_ANTIGEN_ASSEMBLY_WITH_MHC_PROTEIN_COMPLEX | 18 | 1.0942 | 0.033834 | 0.24499 | 4.01E-06 | 0.010770437 | 1.967766656 |
| KEGG_ASTHMA | 27 | 0.83669 | 0.031679 | 0.18622 | 3.54E-06 | 0.010770437 | 1.967766656 |
| KEGG_LEISHMANIA_INFECTION | 65 | 0.5389 | 0.031627 | 0.12125 | 4.43E-06 | 0.010770437 | 1.967766656 |
| GOBP_COGNITION | 299 | 0.23721 | 0.029671 | 0.054268 | 6.22E-06 | 0.013219255 | 1.878793007 |
| GOBP_ANTIGEN_PROCESSING_AND_PRESENTATION_OF_EXOGENOUS_PEPTIDE_ANTIGEN_VIA_MHC_CLASS_II | 29 | 0.83607 | 0.032806 | 0.193 | 7.43E-06 | 0.01403708 | 1.852723214 |
| GOBP_POSITIVE_REGULATION_OF_INFLAMMATORY_RESPONSE_TO_ANTIGENIC_STIMULUS | 12 | 1.1961 | 0.030203 | 0.27924 | 9.26E-06 | 0.015748388 | 1.802763907 |
| GOBP_CELLULAR_RESPONSE_TO_AMMONIUM_ION | 5 | 2.0618 | 0.033613 | 0.48853 | 1.23E-05 | 0.018951551 | 1.722355252 |
| GOMF_MHC_CLASS_II_PROTEIN_COMPLEX_BINDING | 25 | 0.80607 | 0.029369 | 0.19259 | 1.43E-05 | 0.019614149 | 1.707430532 |
| REACTOME_GENERATION_OF_SECOND_MESSENGER_MOLECULES | 30 | 0.80997 | 0.032324 | 0.19402 | 1.50E-05 | 0.019614149 | 1.707430532 |
| GOCC_MHC_PROTEIN_COMPLEX | 23 | 0.87063 | 0.030428 | 0.20961 | 1.64E-05 | 0.019983185 | 1.699335288 |

**
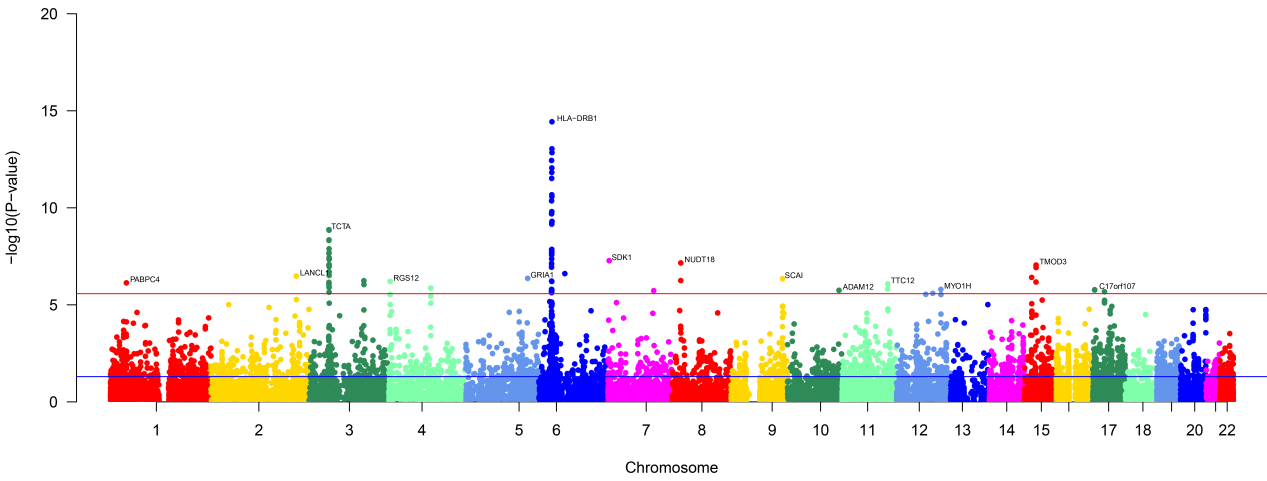
**

**Supplementary Figure1. Gene based for frailty through MAGMA.** Manhattan plot of the cross-tissue transcriptome-wide association results for FI. 437 genes was specifically associated with the risk of FI. The y-axis represents P value in –log(10) scale. A significance threshold after FDR-correction was used.


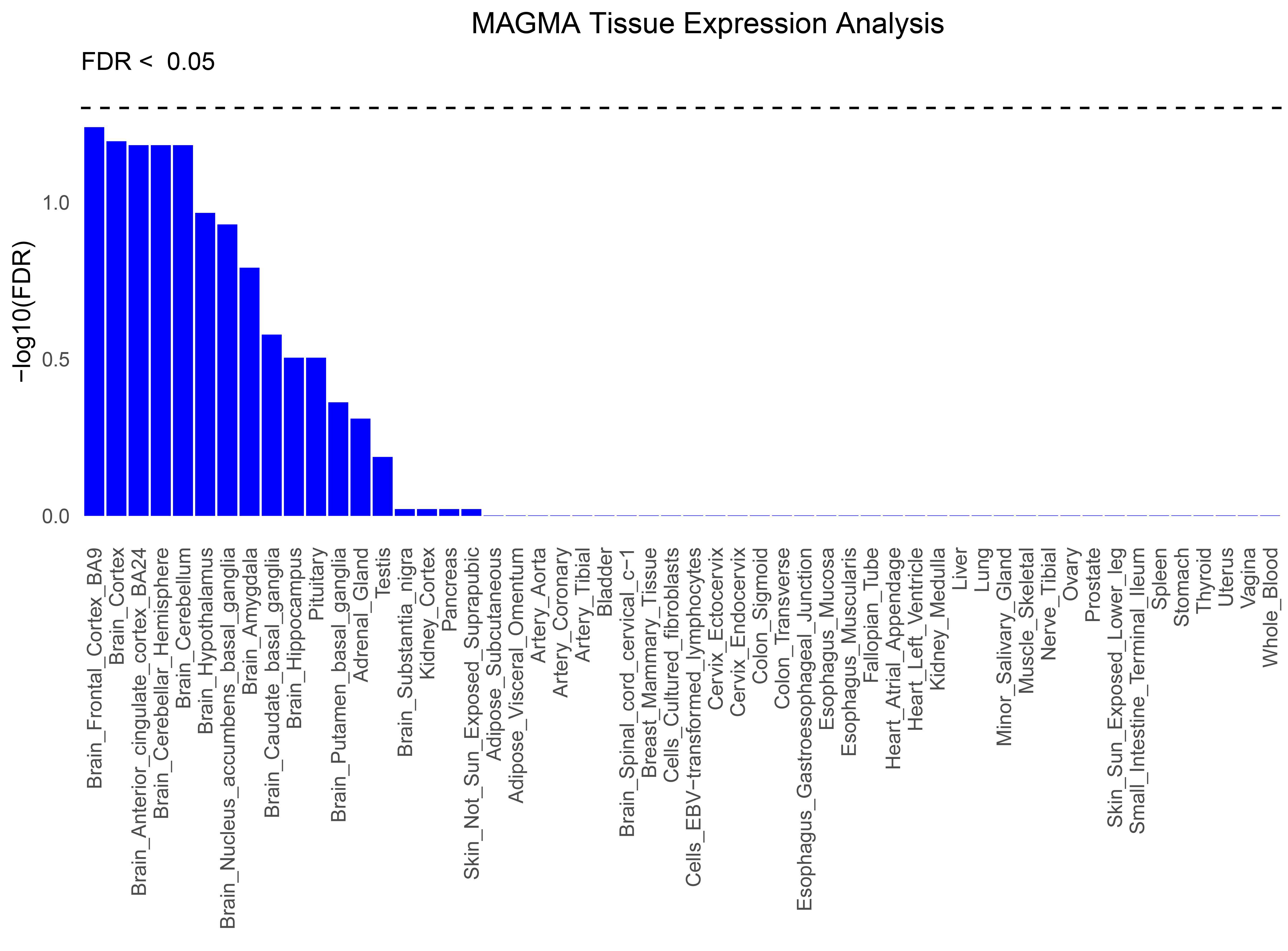


**Supplementary Figure2 Tissue specific enrichment of SNP heritability for frailty through MAGMA.**.

P values are shown in the y-axis with a scale of − log10. The bars in black represent significant enrichment with FDR adjustment for multiple hypothesis testing.
